# Social and Poor Law Problems

**Published:** 1906-05-12

**Authors:** 


### SOCIAL AND POOR LAW PROBLEMS.

### WOMEN AS RELIEVING OFFICERS.

Of late there have been several women appointed as relieving officers, and many boards of guardians are considering the advisability of having at least one woman on their staff for such work. The idea that the work of a relieving officer is of necessity harsh and brutal has long gone out. The appointment of a woman as relieving officer seems to be a natural corollary of the appointment of women as guardians. The latter have found how much there is in Poor-law work that is better done by a woman than by a man. As guardians they can have a direct influence upon the working of the institutions under the control of their board, but they come comparatively little in touch with the out-relief cases, nor is it possible that they can do so except in the rare instances when they can give up their whole time to the work. And even then their position as guardians renders it inexpedient for them to interfere with the work of the relieving officers. But a female relieving officer might in the cases of women and children—and it is these who are the recipients of a great deal of the out-relief given—have a moral power which men cannot possess. One of the Derby guardians in discussing the question, pointed out that a female officer might do much good in the inspection of the homes of boarded-out children. The guardians at Merthyr Tydvil appointed a special committee to inquire into the question of such an appointment, and this committee has laid down what they think should be the duties of a female relieving officer. Among these are: To visit the houses of those receiving relief in kind, to ascertain that the quality and quantity are what they should be (a point which many boards have found to be most uncertain) to guide women to supplement relief by other work, and to find employment for delicate girls; to pay special attention to the aged and imbecile; to see that these were properly lodged, that their clothes were changed weekly, their beds cleaned and precautions taken against vermin; in chronic cases, such as ulcers and abscesses, to see that the affected parts were properly dressed, where professional help was necessary to recommend a nurse, and where speedy recovery was probable to insist that the patients should go to the infirmary for treatment. It is obvious that to fulfil these duties properly the officer herself would need to have some knowledge of nursing. We should think that nurses who have had some experience in district work would be exceptionally well fitted for such posts, and probably many of them would like to have them. It is certainly expected that the female relieving officer should go deeper into things than it is often possible for a man to do.

### SAVED BEFORE BIRTH.

To prevent a high rate of infant mortality you must begin before the child is born. That seems to be the moral of the successful efforts made to save the lives of infants at Villiers-le-Duc, as reported to the French Academy of Medicine. In that village, for a whole century, before the year 1893, the deaths of children under one year had never fallen below 13 per cent., and had sometimes risen as high as 28. In 1892 new measures were taken, with the result that the infantile mortality has gone down to zero. In ten years no child has died under one year of age, and no death has followed a confinement. In Villiers-le-Duc a pregnant woman who does not possess the necessary means to secure, so far as possible, both her own life and that of her expected child, can demand the help from the commune. She is expected to declare her condition before the seventh month, and then she will receive the assistance she needs both before and during her confinement; her child is carefully looked after for a year, and if she produces it in good condition at the end of the year she receives a gratuity of £1 4s. This is certainly an encouragement to good motherhood. On this side of the Channel there is still a strong prejudice against thus taking the burden of parenthood off the shoulders of the parents. When midwifery orders are given by guardians to married women it is usually on loan, on the reasonable assumption that the husband will be able, eventually, if not immediately, to pay the cost of the confinement. That some stringency in the matter is necessary will be readily understood by those who know that young couples—marrying without a penny saved, or even in debt—come to ask for free attendance for the birth of even a first child. If that help were granted without any condition of repayment it is safe to say that all the children who followed would be dependent for their very existence on the parish. For illegitimate children—and the majority of those who need relief before their confinement are unmarried mothers—the maternity wards of the workhouse offer the best provision. Indeed, they would often give more comfort and security even to the married than they are likely to get at home.

